# Rescue from early-onset hearing loss in a mouse model lacking the cyclin-dependent kinase inhibitor p19^Ink4d^

**DOI:** 10.1038/cddis.2016.38

**Published:** 2016-03-10

**Authors:** Q Ma, M Grati, F Bai, J Pei, X-H Pei, X Liu

**Affiliations:** 1 Department of Otolaryngology (D-48), University of Miami Miller School of Medicine, Miami, FL, USA; 2 Molecular Oncology Program, Department of Surgery, University of Miami Miller School of Medicine, Miami, FL, USA; 3 Department of Otolaryngology, Xiangya Hospital, Central South University, Changsha, China

Dear Editor,

Human and mouse genetic-based approaches have been instrumental in identifying key genes and deciphering mechanisms essential for the development, maturation, function and maintenance of auditory organ. In particular, the absence of mammalian inner ear sensory hair cell (HC) regenerative capabilities drove researchers towards gathering clues on their differentiation and maintenance; this would help in the future design of approaches for enhancing HCs' maintenance power and eventually inducing their regeneration.^1^ Mechanisms responsible for cell cycle exit, epithelium patterning and post-mitotic cell cycle arrest of maturing and mature HCs have been a challenging field of focus in auditory research, and the information so far gathered is rather scarce.^2^ For example, in a mouse line lacking cyclin-dependent kinase inhibitor p19^Ink4d^ (p19), Chen *et al.*^3^ have shown that p19 does not influence mouse embryonic stereotyped patterning of cochlea zone of non-proliferating cells into the organ of Corti that is composed of a mosaic of sensory HCs and supporting cells occurring around embryonic days 15.5 and 17.5.^3^ They reported, however, a late progressive loss of sensory HCs accompanied by progressive hearing loss (HL) starting at 2.5 weeks postnatal. We initiated further studies into p19 network regulating HC survival. We detected the presence of p19 starting at postnatal day 3 (P3) and thereafter in cochlea outer (OHCs) and inner HCs (IHCs; Figure 1a). We also generated a new mouse p19 knockout line (*p19^−/^^−^*), where p19 has been entirely deleted.^4^ On these mice, we performed auditory brainstem recordings (ABRs) at P23 that showed a severe to profound HL (Figure 1b). We also used whole-mount immunofluorescence to assess at this stage HC's survival and stereocilium hair bundle morphology, and observed massive death and degeneration of OHCs that are known for their increased vulnerability, and to a lower extent the death of IHCs, accompanied by a generalized severe disorganization of remaining HC stereocilium hair bundles (Figure 1c, P23, green). We looked at the chronology of the HC death and degeneration events, and found that very sporadic HC death at the mid-basal and basal regions of the cochlea started as early as P7 (Figure 1c at P3 and P7, green). Then, HC death has rapidly spread towards the cochlea apex and increased exponentially along all cochlea turns at P12 and thereafter (Figure 1c, P12 and P23, green). However, no vestibular HC death was detectable in *p19^−/−^* mice (not shown). p19, one of the Ink4 family of proteins, exclusively bind to and inhibit cyclin-dependent kinases Cdk4 and Cdk6.^5^ To test the hypothesis if HC death in *p19^−/−^* mice is due to uncontrolled activity of Cdk4, we created double knockout mice *p19^−/−^*;*Cdk4* *^−/−^*.^5^^,^^6^ Indeed, hearing had been perfectly recovered in these mice and ABR thresholds were very comparable at all three tested frequencies to age-matched wild-type mice (Figure 1b) and no HC loss was detectable by immunofluorescence (Figure 1c at P12 and P23, yellow). To our knowledge, this is the first study demonstrating rescuing role of Cdk4 deletion for p19 deficiency *in vivo*. Given the complexity of cell cycle control in mammals, the rescue of p19 loss-induced HC death and HL by *Cdk4* deletion is surprising and demonstrates conclusively the antagonizing functional regulation of Cdk4 and p19 in post-mitotic maintenance *in vivo*. Inappropriate cell cycle re-entry in response to defects in cell cycle control pathways may be a general mechanism through which sensory HCs are damaged or lost. These results further indicate that inhibition of Cdk4 activity by using drugs, such as PD 0332991 that is an FDA approved agent for cancer treatment, could have potential to treat deaf patients caused by aberrant cell cycle re-entry.

## Figures and Tables

**Figure 1:**
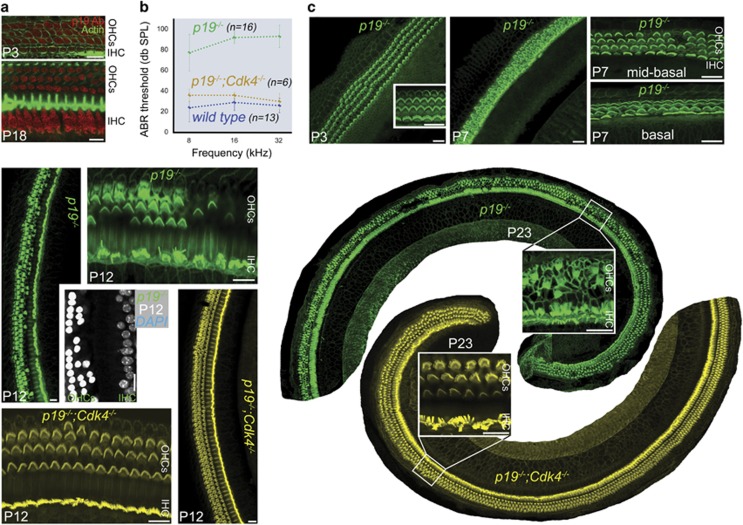
Cdk4 deletion in p19^*−/−*^ mice rescues hair cell (HC) survival and restores auditory function. (**a**) Whole-mount immunofluorescence of postnatal day 3 (P3) and 18 (P18) mouse cochlea using rabbit polyclonal antibody (sc-1063; Santa Cruz Biotechnology, Dallas, TX, USA) showing the early detection of p19 (red) at P3, and thereafter at P18 in all three rows of outer (OHCs) and one row of inner sensory HCs (IHC); actin is counterstained with phalloidin in green. Immunofluorescence experiments were performed as described.^7^ (**b**) Average thresholds of auditory brainstem recordings (ABRs) at pure tone frequencies 8, 16 and 32 kHz on P23-day-old *p19*^*−/−*^ (green; *n*=16) mice and their *p19*^*−/−*^;*Cdk4*^*−/−*^ double knockout (brown; *n*=6) littermates, as well as their age-matched wild-type mice (blue; *n*=13) showing high thresholds at all frequencies in *p19*^*−/−*^ mice synonymous of a severe to profound hearing loss, and much lower thresholds for *p19*^*−/−*^;*Cdk4*^*−/−*^ mice comparable to normal hearing wild-type mice. ABRs were performed as described.^8^ (**c**) Representative low- and high-magnification confocal micrographs of whole-mount immunofluorescence preparations of P3, P7, P12 and P23 cochlea from *p19*^*−/−*^ mice (green), and of P12 and P23 cochlea from *p19*^*−/−*^;*Cdk4*^*−/−*^ mice (yellow). All preparations were stained with phalloidin to reveal actin, with the exception of one DAPI-stained P12 *p19*^*−/−*^mouse cochlea panel (gray). In *p19*^*−/−*^ mice (green): (i) no HC death was detected at P3; (ii) sporadic HC death started to be detected at P7 around the mid-basal and basal turn of the cochlea; (iii) HC death and degeneration, and stereocilium bundle disorganization increased significantly at P12; and (iv) massive death of OHCs and IHCs, and damage of remaining HC stereocilium bundles is observable at P23. In *p19*^*−/−*^;*Cdk4*^*−/−*^ mice (yellow), complete rows of OHCs and IHCs, and normal HC stereocilium bundles have been seen at all stages. Scale bars, 20 *μ*m
